# Automatic diagnostics of tuberculosis using convolutional neural networks analysis of MODS digital images

**DOI:** 10.1371/journal.pone.0212094

**Published:** 2019-02-27

**Authors:** Santiago Lopez-Garnier, Patricia Sheen, Mirko Zimic

**Affiliations:** 1 Unidad de Bioinformática / Laboratorio de Enfermedades Infecciosas, Laboratorio de Investigación y Desarrollo, Facultad de Ciencias y Filosofía—Universidad Peruana Cayetano Heredia, Lima, Peru; 2 Wyss Institute for Biologically Inspired Engineering, Harvard University, Cambridge, Massachusetts, United States of America; Liverpool John Moores University, UNITED KINGDOM

## Abstract

Tuberculosis is an infectious disease that causes ill health and death in millions of people each year worldwide. Timely diagnosis and treatment is key to full patient recovery. The Microscopic Observed Drug Susceptibility (MODS) is a test to diagnose TB infection and drug susceptibility directly from a sputum sample in 7–10 days with a low cost and high sensitivity and specificity, based on the visual recognition of specific growth cording patterns of *M*. *Tuberculosis* in a broth culture. Despite its advantages, MODS is still limited in remote, low resource settings, because it requires permanent and trained technical staff for the image-based diagnostics. Hence, it is important to develop alternative solutions, based on reliable automated analysis and interpretation of MODS cultures. In this study, we trained and evaluated a convolutional neural network (CNN) for automatic interpretation of MODS cultures digital images. The CNN was trained on a dataset of 12,510 MODS positive and negative images obtained from three different laboratories, where it achieved 96.63 +/- 0.35% accuracy, and a sensitivity and specificity ranging from 91% to 99%, when validated across held-out laboratory datasets. The model's learned features resemble visual cues used by expert diagnosticians to interpret MODS cultures, suggesting that our model may have the ability to generalize and scale. It performed robustly when validated across held-out laboratory datasets and can be improved upon with data from new laboratories. This CNN can assist laboratory personnel, in low resource settings, and is a step towards facilitating automated diagnostics access to critical areas in developing countries.

## Introduction

Tuberculosis (TB) is a global and lethal disease, responsible for the ill-health and death of more than 1.4 million deaths each year, ranking above HIV/AIDS as one of the leading causes of death from an infectious disease [1,2]. Timely diagnosis and treatment is key to full patient recovery. About a third of the global population is affected by latent TB infection, and it is believed that around 5–10% of the people develop active TB during their life [3]. Recently, there has been a surge of multi drug resistant TB (MDR-TB), due to lack of early diagnostics, ineffective susceptibility tests and inappropriate treatments [4]. Worse still, we are now facing extreme-drug resistant strains (XDR-TB), which are bacillus with acquired resistance to the most potent anti tuberculous drugs [5,6]. Timely diagnosis, recognition of MDR, and treatment initiation are key to improve patient recovery.

**Fig 1:**
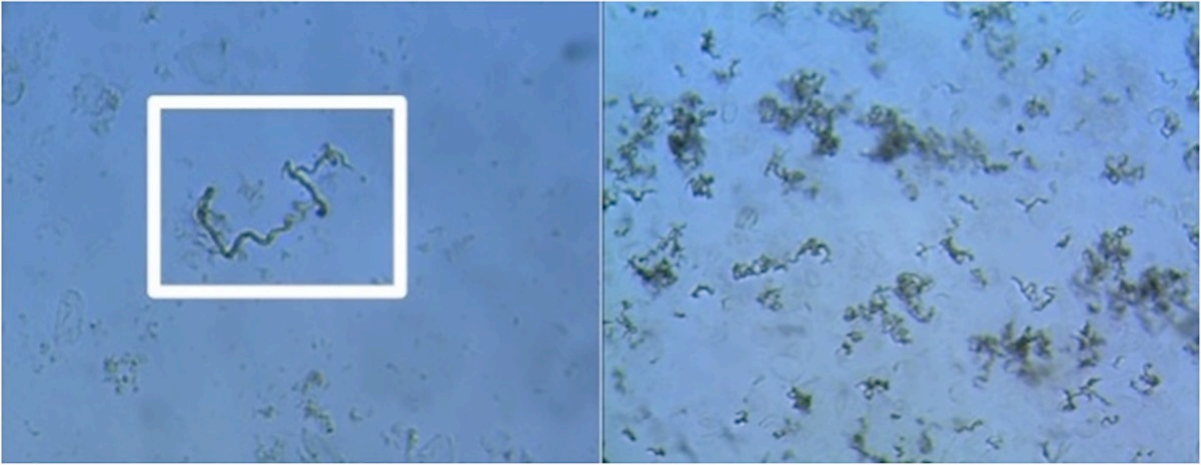
Examples of positive images. On the left, a characteristic M. tuberculosis chord is highlighted in the white box. On the right, a typical image of a positive MODS culture. Notice the chord shaped growth morphology.

In 2011, the WHO began to back a diagnostics test for TB, called MODS (Microscopic Observed Drug Susceptibility) [5,7]. This low-cost method is based on the identification of *M*. *tuberculosis* (MTB) growth in broth in 7–10 days from a sputum sample. A characteristic S shaped, cord-like morphology of colonies in the culture evidences the presence of TB (Fig 1). A typical TB cord in a positive MODS culture exhibits certain morphological and illumination characteristics. A TB positive cord has a particular length and width, and its shape is usually sinuous with a smooth border. Given that a TB cord has a circular transversal section, the light that passes through the diameter shows a high-transmitted brightness, while the light that passes through the border, gets refracted resulting in a lower brightness (Fig 1).

Notably, besides TB detection, MODS is able to simultaneously detect MDR-TB, and XDR-TB with high sensitivity and specificity [7]. Despite its advantages, MODS has a main limitation: the interpretation must be performed by trained personnel, who are often unavailable in low resource settings, which coincidentally are the areas most prone to tuberculosis infections.

In our previous study, a feature-based logistic regression pattern recognition algorithm to automatically interpret MODS digital images was developed [8]. The algorithm used extensive image processing, feature extraction and pattern recognition. It searched for geometrical and illumination features that correspond to those considered by human experts for classification, and was able to detect TB with 99.1% sensitivity and 99.7% specificity [8]. Despite the model’s in-sample accuracy, its performance significantly dropped to 92% when tested on digital images from a held out laboratory. This limitation is caused by the variability in the image's background quality, when samples are processed in different laboratories. Hence, the model required retraining to be deployable at a new laboratory (Zimic M, to be published).

In recent years, advances in machine learning and the implementation of convolutional neural networks (CNNs), paired with the assembly of increasingly complex datasets, have enhanced object classification and detection capabilities [9,10]. CNNs are biologically-inspired hierarchical models capable of making strong assumptions about locality of pixel dependencies in images [11], by learning spatially informed feature hierarchies [10,12]. They consist of feature detector units arranged in layers: lower layers detect simple features and feed into higher layers, which detect more complex features [10,12]. There is compelling evidence that such models can match or outperform human experts on complex tasks, such as image and text processing, recognition and classification [9,10,13–15], decision-making based on abstract representations [16,17], and even decision-making in clinical settings [18,19]. Due to the recent availability of increasingly large medical datasets, it has become possible to develop CNN models for Computer-Aided Diagnosis, and uses in routine clinical applications. These include temporal convolutional networks for disease pattern discovery [20]; detection of glaucoma [21]; detection and analysis of potential breast cancer masses [22,23]; and the classification of skin lesions for the detection of skin cancer [18], amongst others.

Tuberculosis control efforts are hampered by a mismatch in diagnostic technology: modern optimal diagnostic tests are least available in poor areas where they are needed most. Lack of adequate early diagnostics and MDR detection is a critical problem in control efforts.

As explained above, despite MODS being an important test for the diagnostics of TB and MDR, an important limitation that laboratories in the developing world face in MODS implementation is the presence of permanent technical staff with expertise in reading MODS. Based on a dataset of more than 12000 images, we designed and trained a CNN for automatic identification of MTB colonies in a MODS culture. Our computational tool can assist laboratory personnel in MODS interpretation, or substitute for the absence of diagnosticians in low resource settings through telediagnostics.

## Materials and methods

### Dataset composition and structure

#### Primary dataset

Our primary dataset is composed of MODS digital images obtained from the images bank of the Bioinformatics and Molecular Biology Laboratory at UPCH. The MODS cultures and their posterior digitalization were performed in 3 laboratories from the cities of Trujillo, Callao and Lima (UPCH), in Peru, during a previous study (to be published). In that study, each image was assigned a label, positive or negative, by three experts independently. Images in which MTB growth was detected by at least 2 experts were considered positive, and were considered negative if the 3 experts classified them correspondingly. The images were 2048×1536 pixel RGB. To build the dataset, Trujillo provided 9,005 images; Callao provided 5,670 images; and UPCH provided 608 images. After eliminating unreadable images, we ended up with a dataset of 12,510 images: 4,849 positive and 7,661 negative images. Images were rescaled to 224 x 224 pixels, and converted to grayscale. We avoided further image preprocessing, due to the fear of introducing visual artifacts into images of already heterogeneous quality and background (see Fig 1). Additionally, this would allow us to evaluate our CNN’s ability to operate on raw data.

**Fig 2:**
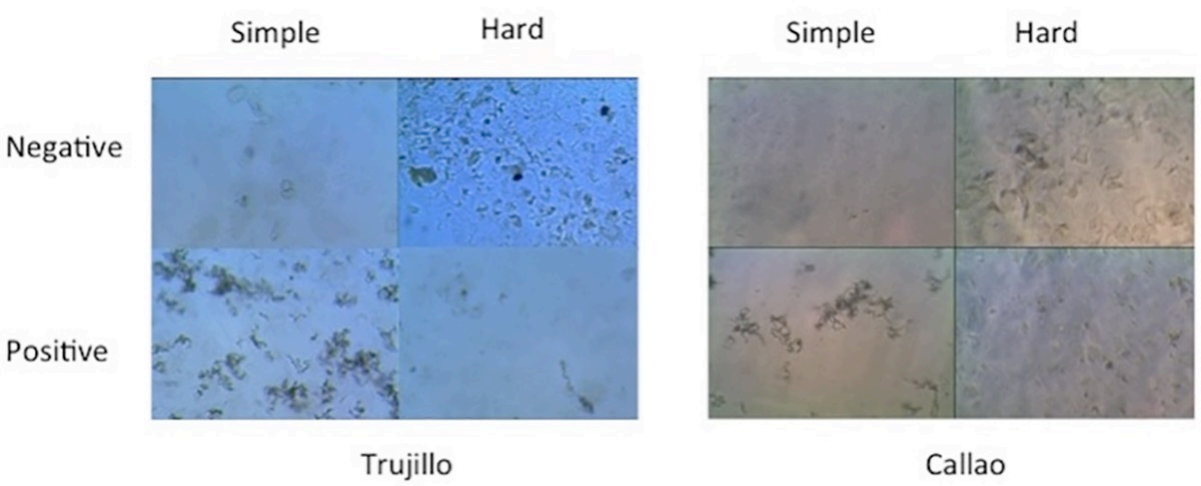
Examples from 2 of the 3 datasets (Trujillo and Callao) of positive and negative frames. We've selected a few images from each dataset to illustrate the difficulty in correctly classifying each image. First column images are from Trujillo; the second column images are from Callao. Notice how similar contaminated negative samples are to relatively clean positive samples.

In order to prevent any overlap or bias between training and validation sets of data, and to allow a robust assessment of the model’s performance with reliable metrics, we performed 5-fold cross-validation. This was achieved by segmenting the original 12,510 image dataset into 5 subsets, composed of 2,502 images each. These subsets were selected randomly from the original dataset, following a 0.63 positive:negative ratio. For each cross-validation step, we used 4 subsets for training/validation (10,008 images), and 1 subset for testing (2,502 images). This allowed the network to train with different images during each round of cross-validation, and to evaluate its performance on unseen images. Models trained on this dataset are referred to as “resc_CNN”. Images corresponding to TB-positive and TB-negative categories, may show different levels of complexity, turning them into images with strong or weak evidence for classification. Fig 2 illustrates the difficulty in correctly classifying images, both in the TB-positive and TB-negative categories.

#### Secondary dataset (paired-laboratory dataset)

In order to demonstrate the model’s robustness and ability to generalize, we trained it on two laboratories worth of images, and had it predict the label of images from a third, held out laboratory’s images. Hence, we generated secondary cross-validation datasets, where we grouped laboratory images in pairs, as follows: group 1 was composed of 8092 images from Callao and Trujillo for the network training, and 484 images from UPCH for validation; group 2, 3400 images from Callao and UPCH for training, and 6004 from Trujillo for validation; group 3, 5518 images from Trujillo and UPCH for training, and 3512 from Callao for validation. These large datasets were then segmented into 5 folds, and 5 cross-validation datasets were generated as described above. Models trained on this dataset are referred to as “2lab_CNN”. Both of these datasets are available for download here.

### Network architecture

**Fig 3:**
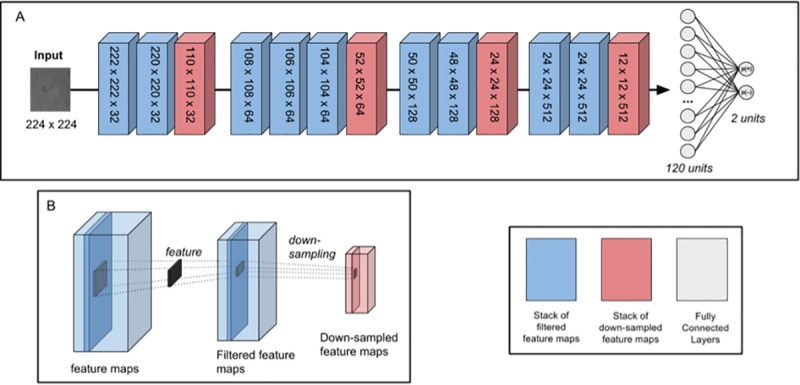
Simplified network architecture. (A) Input to the network is a 224 x 224 grayscale image of a MODS *M*. *tuberculosis* culture. The image is passed through the network, and the output of the second fully-connected layer is a probability distribution over the two classes (positive (+): 1 and negative (–): 0). Each block is a stack of feature maps, of dimensions (width x height x number of feature maps). Layer operations take place between each block (see (B)) and are identifiable by the feature map volume produced. Kernels are 3 x 3 and 2 x 2 for convolutional and pooling layers, respectively. The network is trained and evaluated on a dataset of 1008 train/validation and 2502 test images. (B) A schematic representation of the convolution and pooling operations on an input volume. Note that each convolution operation shown in the figure represents the Conv-BatchNorm-Activation operations.

Our network was designed as a 15-layer deep convolutional neural network, comprising convolutional, max-pooling and fully-connected layers. The CNN architecture is organised in 5 blocks: 4 convolutional and 1 fully-connected/classification blocks, separated by pooling layers (Fig 3). The network was written in Python using Keras high-level neural networks library [24], and used Theano [25] as a backend. The code is available here.

The architecture itself is an adaptation of the VGG16 network, proposed by Simonyan [26], which pioneered the use of deep, multi-layered CNNs with small convolutional filter kernels. This approach has been previously favored, notably by Szegedy’s entry of the ILSVRC-2014 classification task, GoogleNet [9]. It allows the incorporation of multiple non-linear rectification (ReLU) layers in the place of a single one, making the decision function more discriminative. Additionally, it allows for a decrease in the number of parameters to be fine-tuned. We hypothesized that, due to the inherent complexity of our dataset and the need for precise feature extraction, these models were promising templates for experimentation. The architecture was fine-tuned by hyperparameter search and optimization over the basic architecture, and selection of the network that demonstrated best testing accuracy–these iterations can be further explored here.

Input to the network is 1 x 224 x 224, as the images are grayscale. The image dimension was selected because (1) it was the one used by our base model [26], and (2) this was the input that allowed us most flexibility for trying out different variations of filter dimensions and kernel sizes. The conversion to grayscale serves two purposes–firstly, on a practical level, it is less costly in parameters (1 channel vs. 3). Secondly, and perhaps more importantly, we had previously found that certain physical parameters, unrelated to color, were principal predictors of image classification as positive or negative, in a logistic regression model [8]–hence, we reasoned that, if the model were to act as an expert diagnostician and identify similar features (cording characteristics, illumination, edge to surrounding contrast), the variation in color between laboratory datasets would be only be an additional confounding factor, and that we account for it. In other words, we considered that the information we would lose by reducing channels would probably be a source of noise, rather than an important feature for classification.

All of the convolutional layers apply 3 x 3 kernels with stride = 1 to the input volume, mapping features to activation map stacks in deeper layers of the network. This kernel dimension was selected because it allowed to stack more convolution operations per block [27], to obtain a larger, locally dependent receptive field which provide more expressive features [28]. The receptive field of 3 x 3 is the smallest to still capture notions of directionality [26]. This small receptive field convolves the input at each convolutional layer, and allows stacking of convolutional operations. These smaller sized filters have also been used previously by Ciresan et al. [13]. In practice, stacking two 3 x 3 convolutional layers results in an effective receptive field of 5 x 5 [26]. This is interesting, because using two 3 x 3 convolutional layers instead of one 5 x 5 layer allows (1) the incorporation of 2 ReLU activation layers, that make the decision function more discriminative and (2) a decrease in the number of parameters [26]

As the inputs to the convolutional layers do not have spatial padding, there is an effective reduction of 2 pixels in width and height per convolution operation, helping to reduce activation map volumes and therefore the number of parameters optimized. This, paired with spatial pooling (see below) was partly to compensate for the increase in number of tunable parameters due to network depth, the convolutional layers, The kernels’ outputs are activation map volumes of dimension (filtered image width x filtered image height x number of filters generated). Because in deeper layers, features become more complex, we doubled the number of filters optimized by each convolutional layer, between each block of layers. As these features become more complex, they map onto larger regions of the input volume, making their role in identifying features in the input data fundamental.

To further allow for feature extraction, we configured the CNN’s 4^th^ block, comprised by two convolutional layers, to output the same dimensions as their inputs. This maintained the activation map volume at 512 x 24 x 24. This allowed for the filtering over a conserved input volume. Hence, it permits the extraction of more meaningful features from the input [11].

Batch normalisation was used prior to ReLU activation. In our CNN, batch normalisation allowed us to use higher learning rates for network optimisation without the risk of divergence, regardless of weight initialisations; it also accelerates the training of deep neural networks [29]. ReLU nonlinearity also allow for faster training of CNNs, which helps to avoid large models overfitting on smaller datasets [11,27].

Spatial pooling is carried out by 4 max-pooling layers, one at the end of each layer block. Max-pooling operations were performed using 2 x 2 kernels with a stride = 2, as recommended previously [13,26]. This kernel dimension allowed the network to reduce the spatial size of the representation by eliminating non-maximal values, which in turn reduced the number of parameters and computational time; additionally, it helped to control overfitting by providing a form of translation invariance [13,26].

Finally, the fourth convolutional block is followed by two fully-connected layers. The first has 120 units. The second, sigmoid layer, is composed of two units (one per category), that outputs a probability distribution over the two categories (positive (+): 1 and negative (–): 0).

### Training

During the training phase, we provide the model with images belonging to each cross-validation training dataset. Prior to training, we split the training data into training and validation data. The validation data allowed us to monitor our model's performance and select the best model for later testing and performance evaluation. The split was performed to keep 85% of the data for training, and reserve 15% for validation. To increase the number of training samples, we used Keras’ data augmentation feature. This generated batches of image data with real-time data augmentation, useful to avoid over-fitting and enhance evaluation of the network’s performance, while keeping the original label (positive or negative) of each image [11,13]. The modifications performed on the original images consisted of rotations, width and height shifts (1/10 of the original image), as well as horizontal and vertical flipping of the images. We used He Normal weight initialization, which facilitates convergence, even in very deep convolutional networks [14]. We used Keras' checkpoint feature to create checkpoints of the model's parameters after the training epoch during which our model's validation accuracy was highest. After each training session, a new model instance was called, which reset the previously optimized parameters, and the newly instantiated model was trained on the next dataset. This allowed for objective assessment of the model's reported metrics. In this sense, it is important to note that our models were not trained across all cross-validation datasets: each model trained on a certain fold of the cross-validation datasets (for example, training data from dataset 1) was fine-tuned and tested only with its corresponding test data (test data from dataset 1).

The metric monitored to guide the network towards minimal error loss and create checkpoints was binary cross-entropy loss, calculated by the binary cross-entropy cost function (logloss). For hyperparameter selection, we first performed a hyperparameter search, exhaustively trying combinations of hyperparameters (learning rate, optimizer, batch size and dropout). From this hyperparameter search, we selected the model that demonstrated highest validation metrics. Hence, we ended up using RMSProp with a learning rate = 10^−3^ as the optimizer to minimize the cross entropy loss. The network was trained for 100 epochs, using a batch size of 72, and dropout of 0.5. Dropout prevents units from co-adapting and forces them to learn more robust features by setting to zero the output of a given hidden unit, with a probability of 0.5 [11,30]. The training was conducted on a NVIDIA GeForce GTX TITAN X (12 Gb DDR5 RAM, 512 bits, 3072 cores) GPU. The training lasted 3.5 days for the resc_CNN, at 700 s per epoch. After the initial training, the network was fine-tuned for 100 more epochs, with RMSprop and a learning rate = 3 × 10^−4^.

To evaluate the model’s robustness, we performed two rounds of training: first, we trained the resc_CNN network with the parameters as described above, on the secondary datasets. After a first round of 100 epochs on 5 sub-datasets per pair of laboratories, we obtained five sets of weights per pair of laboratories. We validated the network on the held out laboratory’s images, and selected the set of weights per laboratory pair that allowed for highest validation sensitivity. The second round of training consisted in using these weights to generate new instances of the model, per cross-validation split and per pair of laboratories, and trained the networks for 100 more epochs.

### Network performance validation and metrics

To evaluate the classification performance of our network, we used 5 metrics: the binary accuracy, sensitivity, specificity, Matthews Correlation Coefficient (MCC) and F1 score. The binary accuracy calculates the mean accuracy rate across all predictions for binary classification problems. The sensitivity (or recall) is the fraction of truly positive images that the algorithms classifies positive. The specificity (or true negative rate) is the fraction of truly negative images that the algorithm classifies as negative. The Matthews Correlation Coefficient is a metric used to evaluate the quality of binary classifications. Lastly, the F1 score is a metric that combines precision and recall, relative to the positive (TB) class.

As mentioned previously, we opted to separate training and testing steps, to allow for simultaneous model training and testing. To evaluate our network’s performance, we loaded the best model saved by the checkpoint during training, and validated it on unseen images. This validation set varies amongst datasets, and allowed us to perform five cross-validation for the resc_CNN models. The mean and standard deviation for each metric were then calculated.

To demonstrate the model’s robustness, we loaded the best model saved by the checkpoint feature during the secondary training step on the paired-laboratory dataset of images. Here, each model instance corresponds to the best model generated by a certain fold of cross-validation training on images from a pair of laboratories. The validation images were from the held out laboratory, hence resulting in three 2lab_CNN models, one per paired-laboratory datasets. The mean and standard deviation for each of the aforementioned metrics on each paired-laboratory datasets were then calculated.

In addition to evaluating the model's performance, we were interested in visualising the features that the model's convolutional layers were optimising. This provides insight into the function of intermediate feature layers, and the evolution of features during training [28]. We achieved this using the filter visualisation script, adapted from Keras’ visualization utility. This method relies on maximizing the activation of filters in specific convolutional layers of our model’s architecture, after training [31]. Starting with a grayscale image (x), the activation (a(x)) caused by the input is computed by a loss function, and 100 steps are taken in the input space along the gradient (gradient ascent) to modify the input to cause higher activations [32].

The filters are sorted by descending activations, and the best 49 filters are saved.

Additionally to visualizing features, we synthetically generated input images that would maximize the activation for a specific class–in particular, we were interested in evaluating how the features deemed most useful in label attribution by the model relate to these synthetically generated positive images. To achieve this, we generated negative and positive images that maximized the activations of the 0^th^ and 1^st^ neurons respectively, in our output classification layer. This is done similarly to the filter visualization procedure: gradient ascent was performed for 2000 steps on noisy gray images, and the top 9 images, sorted by activation, were saved. Here, the activation is the probability assigned by the model’s output layer to a generated input that it belongs to the corresponding index (0 or 1) [31].

We were also interested in assessing the possible similarities between features learned by our 3 models generated by the 2-laboratory training / 1 laboratory validation datasets. This was also performed as described above, and we also generated images of positive class for comparison. The generated images, filters, and model parameters are available for download here.

## Results and discussion

### Network performance

Resc_CNN was trained and tested independently on 5 datasets generated by cross-validation, each composed of 12510 images (10008:2502 training/validation split—data). In each fold, a different fifth of the total dataset is used for testing. The mean and standard deviation from the validation metrics across all 5 of cross-validation were computed over the corresponding testing dataset (Table 1). Our CNN achieved 96.63 +/- 0.35% (mean s.d.) binary accuracy, 94.74 +/- 0.89% sensitivity and 97.83 +/- 1.07% specificity.

**Table 1:** Cross-validation results for the resc_CNN model.

model	cross-validation folds	Binary Accuracy	Sensitivity (Recall)	Specificity	MCC	F1 score
resc_CNN	5	96.63 +/- 0.35%	94.74 +/- 0.89%	97.83 +/- 1.07%	0.8986 +/- 0.0045	0.9562 +/- 0.0041

Despite the reduced training dataset size, and the difference in quality between images in the training and validation datasets, our three models were able to identify tuberculosis on a held out dataset, with an average 95.76% accuracy, 94.27% sensitivity and 96.81% specificity (Table 2).

**Table 2:** Cross-validation results for the 2lab_CNN model.

model	cross-validation folds	Binary Accuracy	Sensitivity (Recall)	Specificity	MCC	F1 score
2lab_CNN_1	5	99.01 +/- 0.62%	98.60 +/- 1.31%	99.38 +/- 0.77%	0.9681 +/- 0.0223	0.9894 +/- 0.0066
2lab_CNN_2	5	93.24 +/- 1.98%	91.12 +/- 2.34%	94.36 +/- 3.00%	0.8152 +/- 0.0418	0.9022 +/- 0.0265
2lab_CNN_3	5	95.03 +/- 1.14%	93.08 +/- 2.45%	96.69 +/- 1.22%	0.8486 +/- 0.0383	0.9448 +/- 0.0131

**Fig 4:**
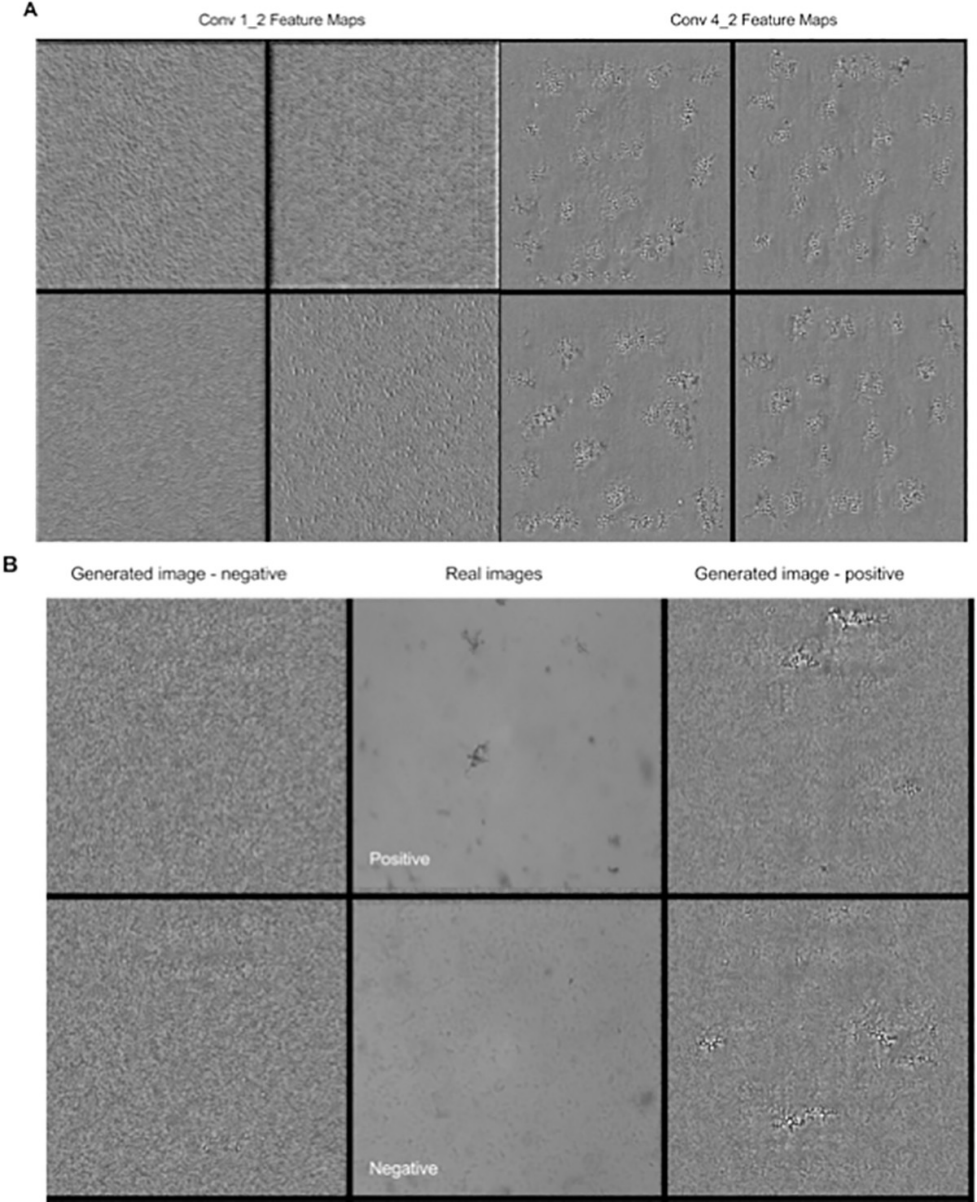
Features learnt and images synthetically generated by resc_CNN. (A) Visualisation of features and comparison of the learnt by convolutional layers. We randomly selected the features that caused top 4 activations across all feature maps of the 2^nd^ and 9^th^ convolutional layers. (B) Synthetically generated images. Images generated by gradient ascent maximize the activation out of the *j*^*th*^ layer of the CNN, for the positive and negative classes, alongside examples of positive and negative testing images, as provided to the CNN for classification. The figure is best viewed in digital format, and the full collection of generated features and synthetic images are available here.

Furthermore, in order to identify the features learned by the CNN, we proceeded to visualisation of the features that the model's convolutional layers were optimizing (Fig 4A).

**Fig 5:**
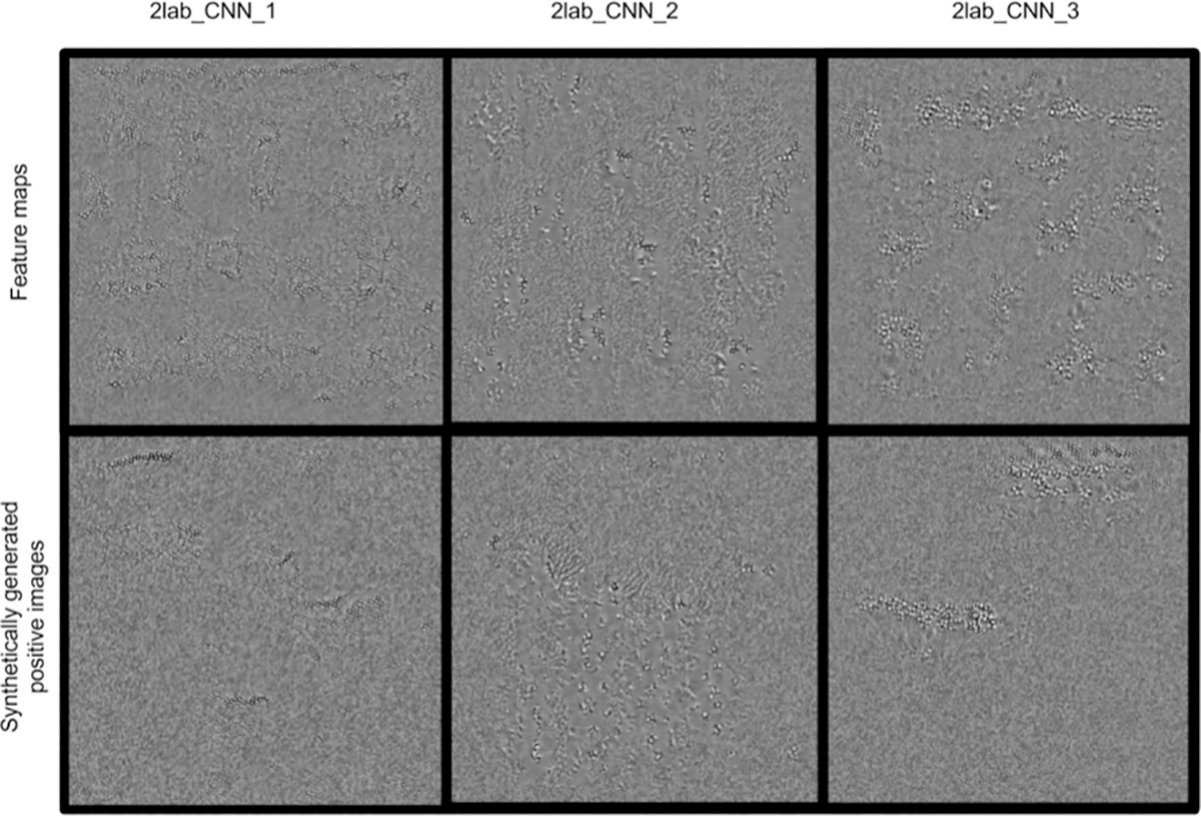
Features learnt and images synthetically generated by the 2lab_CNN model. Top: features extracted from model trained on the three different datasets; bottom: synthetically generated positive images, as mentioned above.

In addition to extracting features from the resc_CNN, trained and validated on data from three laboratories, we proceeded to compare features from the last convolutional layer of the three 2lab_CNN models (Fig 5). Notably, The features extracted from the three models trained on different pairs of data are similar, and resemble the features extracted from the resc_CNN model.

However, these results are insufficient to claim that the CNN actively uses the features to classify the test images, or that these features are deemed sufficient by the CNN to assign class labels. Therefore, we proceeded to generate synthetic images by gradient ascent, which maximize the activation out of the j^th^ layer of the CNN. This image generation can be performed both for the positive and negative classes. For means of comparison, we have also provided real images corresponding to each class for comparison (Fig 4B and Fig 5). The features identified as most useful for classification are visible in the generated positive image.

This study shows that a CNN can be trained ad-hoc to interpret MODS cultures digital images for diagnostics of TB. This network can correctly classify MODS images with high accuracy, sensitivity and specificity.

When evaluating our model’s ability to generalise, we found that when laboratory pair datasets were validated with a third, held out laboratory dataset, the model’s metrics were similar to what it achieved after training on the whole, shuffled dataset. The UPCH laboratory has the most experience in digitalizing MODS cultures, so images provided by this laboratory are of the highest quality. Intermediate experience and image quality correspond to the Callao laboratory, followed by the Trujillo laboratory, which had just begun to perform MODS cultures during the execution of this study. The three models tested achieved ≥90% sensitivity and specificity, despite the reduced training dataset size, and the difference in quality between images in the training and validation datasets (Table 2).

Furthermore, we found that the model's learned features resemble visual cues used by expert diagnosticians to identify MODS cording patterns. Within these features, the cording shapes and edges are identifiable, and these resemble *M*. *tuberculosis’* characteristic growth in MODS culture (Fig 4A). The first convolutional layer appears to extract textures, both from the background of the images and of the TB colonies. The deeper convolutional layers, in turn, detect cord-shaped elements, which are more abstract, high level features.

We have shown that by training the model on two laboratory datasets and using a third laboratory dataset as a validation set, the model generates features that are practically indistinguishable from one laboratory pair to another. This suggests that the model optimises features that are general across laboratories, and supports our hypothesis that our model can generalise and scale on larger datasets and different settings.

Finally, not only do the features from models trained on different laboratory datasets resemble each other: by generating synthetic positive and negative images, we have shown that these features can also be identified in the synthetically generated positive images, which supports the hypothesis that the features are essential to the model’s classification. Within the generated images, cord-like elements are identifiable. They appear spaced out, and seem to be surrounded by a lighter region. This brightness variation creates contrasts between the background and the features themselves. After closer inspection of the positive training images, this illumination change is visible but less evident than in the synthetically generated imagines, which would appear to indicate that the CNN considers it as an important parameter for adequate classification. It is also interesting to note that our previous study had identified this parameter (illumination characteristics) as well as the geometric cording characteristics as being the main predictors of images belonging to the positive class, in our logistic regression model [8]. These features are especially visible when extracted from the model trained on the whole dataset (resc_CNN). This would indicate that the model’s performance is closely related to the optimization of the extracted features, as is the case with human expert diagnosticians.

With regards to our models trained on held-out datasets, even in comparatively small datasets (for e.g., paired-dataset 2), where the number of training samples represents only half of the number of validation samples, the model achieved about 91% and 94% sensitivity and specificity, respectively. This, as well as the low standard deviations across the five testing sets, suggests that our CNN’s features have converged.

It is also noteworthy that the 2lab_CNN model that performed best (2lab_CNN_1) had been trained on 8092 low quality images from Callao and Trujillo, and validated on high quality images from UPCH. The two other models performed somewhat worse, despite having been partly trained on high quality images from UPCH, where the training set was of 3400 and 5518 images for models 2 and 3, respectively. Altogether, this data suggests that although the data quality affects the model’s ability to learn features, the amount of training data available is an important limiting factor for model optimization. Hence, to perform a fair and definitive assessment of the model’s robustness, a larger and more balanced dataset of images will be required.

Prior to the use of CNNs, there have been efforts devoted to the development of machine-learning tools to facilitate TB diagnostics [33–36]. These were intended to palliate the lack of radiology and microbiological interpretation expertise in many underserved areas of the world. These tools have often been used to detect pulmonary TB from chest radiographies [33–36], and less frequently, directly from sputum samples.

Diagnostics based on chest radiographies rely on posterior anterior chest radiographies, which various machine-learning tools can examine for patterns suggestive of TB [34,35]. Additional information, such as symptoms, can also be included as part of the algorithm’s inputs [34]. For the sake of brevity, we note a few examples; readers are directed to by Jaeger et al. [35] for a broader survey of alternative approaches to chest radiography examinations.

Melendez et al. [34] use random forests and extremely randomized trees methods, in combination with clinical information, and achieve 95% sensitivity and 49% specificity. However, these metrics deteriorate when the methods are used separately [34]. Additionally, the combined data-driven approach may influence the diagnostic having clinical information outweigh the image-based interpretation, which could be a result of the training dataset [34]. Xu et al. [36] developed a TB cavity detection system, based on a support vector machine (SVM) for coarse feature classification, a Gaussian model-based template matching, amongst other tools. Despite the extensive image enhancement for feature extraction, the peak sensitivity and specificity are 78.8% and 86.8%, respectively [36]. Other authors have also reported the use SVMs for automated detection of TB, with some success [37].

Melendez et al. [38] recently presented a method of computer-aided detection (CAD) that assists in triage of individuals with suspected active pulmonary TB. When evaluated, it yielded 55.71% specificity at 95% sensitivity, but on dataset comprising of only 87 positive cases, of which 61 were confirmed. Although it is a promising alternative for triage in low TB burden settings, its use in low resource settings is questionable, where access to X-ray equipment might be challenging, and microbiological assays may be more accessible.

Another study by Cao et al. [39] uses an adaptation of the GoogleNet CNN model, pretrained on the ImageNet dataset and fine tuned on 4701 chest X-Ray images, to achieve 89,6% binary accuracy. It is conceivable that their fine-tuning dataset was insufficient to achieve higher accuracy. There is also no reporting of other relevant metrics. Another study by Cao et al. [40], also using both AlexNet and GoogleNet, finds similar binary accuracy. Here, the Precision, Recall, and F1 score are calculated, and vary quite considerably between validation folds. Additionally, the model is trained on less than 5000 images, and without data augmentation. It is conceivable that, due to large model size, it is simply overfitting. Validation on a held-out dataset, as we performed on our 2lab_CNN models, is necessary.

Lakhani et al. [33] also use CNNs to analyze chest radiographs, and as did Cao et al., use AlexNet and GoogleNet. Both models demonstrate noteworthy metrics–GoogleNet achieves 92% sensitivity and 98.7% specificity, while AlexNet achieves 92% sensitivity and 94.7% specificity. The performance is similar when both models’ decisions are taken into account, but the performance is notably boosted when the diagnostic is Radiologist-augmented. Although the metrics are impressive, here too the model was trained on limited data– 1007 total patients, 150 used for testing (75/75 positive/negative). Additionally, there was a lack of validation on a held-out laboratory dataset, although the authors did have 4 independent datasets to work with, which would have provided interesting insight as to their models’ ability to generalize.

Lopes et al. [41] provide an interesting attempt to benchmark the performance of some famous CNN architectures on 4 TB datasets, individually and as methods of feature extraction for posterior TB diagnostics by a SVM. The data used was from public datasets of X-ray images. The best accuracies were obtained from models that combined feature extraction by the CNN (GoogleNet / ResNet / VggNet) and classification by SVM, and range from 76,2% to 84,7%. These metrics are also inferior to ours, and this might be explained by the reduced size of available datasets used for benchmarking.

Hwang et al. [42] base their CNN model on AlexNet, and similarly to the previous studies, use transfer learning for model initialization. They use 3 datasets to evaluate their model, and achieve 90% accuracy across these datasets. Interestingly, they perform a “cross-dataset” experiment, which is analogous to our experiments with models trained on held out datasets. However, their accuracies fall to 83%, while we have shown almost identical metrics between our general model to our held-out dataset models. This study is interesting, and differs from the previous ones, in that it uses a relatively large dataset (more than 10’000 images), which might explain the model’s laudable performance.

Jaeger et al. [35] note a few issues with diagnostic tools based on the analysis of radiography images: there is a lack of a common dataset, which in turn causes the inability to compare classifiers; these approaches are inherently indirect diagnostic methods, and may require confirmation through biochemical or visual methods; they often may require extensive preprocessing (for instance, bone suppression, or lung boundary detection); and lastly, the characteristics used for image analysis are often present in other diseases [35]. We would also suggest that it is debatable whether a chest radiography approach would truly be more available in low resource settings, or remote communities.

A less common approach is the direct identification of TB in growth medium, often through recognition of is distinctive growth characteristics. In fact, we were unable to find peer-reviewed, published references that made use of this alternative. We have argued, as was detailed in the previous sections, that our approach harnesses the inherent strengths of CNNs, while also avoiding the pitfalls of extensive image processing, feature selection, and other confounding factors.

In addition to the aforementioned advantages that CNNs have over other machine-learning methods, it has been suggested that deep networks are robust to label noise [43–45]. Some models perform better on non-curated datasets [46]. There is also evidence that CNNs are robust to label noise that is spread randomly across training sets [47]. However, this is controversial, and still to be studied extensively. It is important to highlight that, although different approaches are available for TB diagnostics, these have not been developed specifically for MODS culture image analysis, which is the main interest of this study, given the importance of MODS as explained above.

Amongst the methods that utilize microbiological image analysis, is our previous attempt at an algorithm for MODS interpretation, based on a logistic model classification of extracted features. This algorithm achieved 99.1% sensitivity and 99.7% specificity [8]. However, these metrics were estimated on images of the UPCH laboratory and after extensive image processing. This high quality dataset of images is not fully representative of the images obtainable during field-validation. We observed that the morphological / illumination characteristics of the background on the MODS digital images varied considerably within the 3 mentioned laboratories. This is probably due to the variability in the microbiological processing of the samples. Due to the aforementioned reasons, this approach is limited to functioning site-specifically. Therefore, this algorithm needs to be re-trained each time it is to be used at a different laboratory, with data provided in advance.

To avoid these issues, we trained our CNN on images provided by 3 different laboratories. This allows for features to be selected according to their capacity to help identify MTB growth, regardless of its site of origin. This is possible because of the intrinsic property of CNN convolutional layers to generate and optimise learnable high-level features [10–12], fine-tuned according to their capacity to perform the required classification at scale.

The aforementioned approaches are encouraging and represent significant advances in providing access to affordable, timely TB diagnostics, but have inherent limitations, which our method lacks. Briefly, diagnostics tools based on radiography images are frequently dependent on TB confirmation through other methods, often require extensive image preprocessing, and it is also improbable that the equipment to perform X-Ray radiographies are available in low resource settings. Alternative microbiological sample-based diagnostics have also been devised, but their application is limited by algorithms that necessitate laboratory-based model re-training. Hence, we consider our approach to be more suitable in low resource settings, as facilitates the coordination of an accessible and rapid TB culture strategy, in MODS, with a high performance telediagnostics method, in our CNN. We have already shown that this coordination performs well, with the implementation of a Web-based platform–the particular feature allowing for logistic regression-based TB diagnostics is available here: http://www.upch.edu.pe/bioinformatic/portal/tuberculosis/.

We believe that, as we collect more data and train the general model, its robustness will become sufficient to overcome the need for laboratory-specific retraining.

We anticipate that this tool will be key in facilitating access to quick and reliable diagnostic of tuberculosis, and are currently exploring directions in which further work is needed. For instance, the further optimization of the architecture; the training of the reference model on larger and more balanced datasets of MODS digital images; and the possible implementation of a real-time, video monitoring system of MODS samples for possible growth of MTB.

## Conclusions

We have demonstrated the effectiveness of our system for automatic diagnosis of TB, using a CNN trained on 12510 images of 7–10 days MODS cultured samples, both with and without the growth presence of MTB. The images were obtained from three different laboratories, and despite their considerable variability, the CNN achieved 96.63 +/- 0.35% accuracy, 94.74 +/- 0.89% sensitivity and 97.83 +/- 1.07% specificity. Importantly, it performed robustly when validated across held-out laboratory datasets, and showing metrics comparable to those of the model trained on the whole dataset. We anticipate that the model will be improved upon with data from new laboratories. The model's learned features resemble visual cues used by expert diagnosticians to interpret MODS cultures, suggesting that our model may have the ability to generalize and scale. We hope that the system will facilitate access to a reliable, timely diagnostic of TB, especially needed in low-resource settings.
